# EPAS1, a hypoxia‐ and ferroptosis‐related gene, promotes malignant behaviour of cervical cancer by ceRNA and super‐enhancer

**DOI:** 10.1111/jcmm.18361

**Published:** 2024-05-09

**Authors:** Xiaoqin Lu, Wenyi Zhang, Jingyan Zhang, Dan Ren, Panpan Zhao, Yanqi Ying

**Affiliations:** ^1^ Department of Obstetrics and Gynecology The Second Affiliated Hospital of Zhengzhou University Zhengzhou China

**Keywords:** cervical cancer, EPAS1, ferroptosis, hypoxia, malignant behaviour, super‐enhancer

## Abstract

Hypoxia and Ferroptosis are associated with the malignant behaviour of cervical cancer. Endothelial PAS domain‐containing protein 1 (EPAS1) contributes to the progression of cervical cancer. EPAS1 plays important roles in hypoxia and ferroptosis. Using the GEO dataset, machine‐learning algorithms were used to screen for hypoxia‐ and ferroptosis‐related genes (HFRGs) in cervical cancer. EPAS1 was identified as the hub gene. qPCR and WB were used to investigate the expression of EPAS1 in normal and cervical cancer tissues. The proliferation, invasion and migration of EPAS1 cells in HeLa and SiHa cell lines were detected using CCK8, transwell and wound healing assays, respectively. Apoptosis was detected by flow cytometry. A dual‐luciferase assay was used to analyse the MALAT1‐miR‐182‐5P‐EPAS1 mRNA axis and core promoter elements of the super‐enhancer. EPAS1 was significantly overexpressed in cervical cancer tissues. EPAS1 could increase the proliferation, invasion, migration of HeLa and SiHa cells and reduce the apoptosis of HeLa and SiHa cell. According to the double‐luciferase assay, EPAS1 expression was regulated by the MALAT1‐Mir‐182‐5p‐EPAS1 mRNA axis. EPAS1 is associated with super‐enhancers. Double‐luciferase assay showed that the core elements of the super‐enhancer were E1 and E3. EPAS1, an HFRG, is significantly overexpressed in cervical cancer. EPAS1 promotes malignant behaviour of cervical cancer cells. EPAS1 expression is regulated by super‐enhancers and the MALAT1‐miR‐182‐5P‐ EPAS1 mRNA axis. EPAS1 may be a target for the diagnosis and treatment of cervical cancer.

## 1 INTROUDUCTION

Cervical cancer (CC) is the fourth most common malignant tumour among women in the world, accounting for 4% of all malignant tumours. A previous study suggests that every year, about 527,624 women are diagnosed with CC and about 265,672 women die from it.^1^ CC has now become one of the malignant tumours that pose a severe threat to the lives and health of women.

A key feature that affects the development, metastasis and prognosis of cervical cancer is the presence of a hypoxic microenvironment. The rapid proliferation of tumours exceeds the capacity of the surrounding vascular system, leading to a decrease in oxygen levels within the tissue and causing hypoxia. Thus, areas with low oxygen levels are formed, commonly known as hypoxic areas.^2^ Hypoxia generates an oxygen gradient in the tumour tissue, affecting tumour plasticity and heterogeneity and promoting tumour invasiveness and metastasis. Hypoxia‐inducible factor (HIF), a key regulator of the hypoxic response, is the core factor involved in oxygen supply changes.^3^, ^4^, ^5^ HIFs induce chronic (continuous, non‐interrupted) hypoxia in tumour microenvironment (TME).^6^ In the progression and treatment resistance of cancer, HIFs is fully involved in every hallmark of cancer, especially in the two processes that rely on oxygen, angiogenesis and metabolic reprogramming.^7^ The interaction between HIFs and various pro‐inflammatory factors derived from tumour cells affects the angiogenesis in cancer tissues.^7^, ^8^, ^9^ HIFs regulate the metabolic reprogramming of cancers, including glucose metabolism, lactate and acidification, lipid metabolism and amino acid metabolism.^10^, ^11^, ^12^, ^13^ Hypoxia‐inducible factors can also affect tumour metabolism by regulating mitochondrial function.^14^, ^15^, ^16^, ^17^ Moreover, regardless of the treatment method, hypoxia is associated with poor prognosis in cervical cancer.^18^, ^19^ Clarifying the expression and regulatory mechanisms of HIF is of great significance for the diagnosis and treatment of cervical cancer.^20^

Ferroptosis is a reactive oxygen species (ROS)‐dependent form of cell death, characterized by iron accumulation and lipid peroxidation. The ferroptosis activator, erastin, inhibits the antioxidant system by increasing intracellular iron accumulation. Iron generates excess ROS through the Fenton reaction, leading to oxidative damage. lipoxygenase (ALOX) and EGLN prolyl hydroxylases (also known as PHD) are the main enzymes responsible for lipid peroxidation and oxygen homeostasis. Iron can affect iron‐mediated death by regulating the activities of ALOX and PHD.^5^, ^21^, ^22^ Lipid peroxidation mainly affects unsaturated fatty acids in the cell membrane. As a free radical‐driven reaction, lipid peroxidation products, such as initial lipid hydroperoxides (LOOH), malondialdehyde (MDA) and 4‐hydroxynonanal (4HNE), increase during ferroptosis.^23^, ^24^ Ferroptosis is an adaptive process that is crucial for eliminating cancer cells.^25^ Furthermore, ferroptosis is involved in the occurrence and development of cervical cancer and is related to its prognosis.^26^, ^27^, ^28^, ^29^, ^30^ Some drugs exert therapeutic effects against cervical cancer through ferroptosis.^31^, ^32^, ^33^ Hypoxia reduces Fenton chemistry and lipoxygenase activity, which are the key control systems for ferroptosis.^34^

Super‐enhancer is a large cluster of transcriptionally active enhancers, which refers to super‐long cis‐acting elements that are 8–20 kb in length with transcriptional enhancer activity and can assemble high‐density key transcription factors and their cofactors. The differences between super‐enhancers and ordinary enhancers are as follows: (1) Super‐enhancers are highly modified by H3K27ac and H3K4me1 and bind mediator complexes to the BRD4 protein. (2) The transcription factors bound by super‐enhancers and the transcriptional activity markers on chromosomes are much higher than those bound by ordinary enhancers. (3) Genes regulated by super‐enhancers have much higher expression levels than genes regulated by ordinary enhancers. (4) The single enhancer that makes up a super‐enhancer can activate gene transcription like the normal enhancer.(5) Super‐enhancers can bind specific transcription factors in tissues. (6) Compared with normal enhancers, blocking transcription factors are more sensitive to the effect of super‐enhancer activity.^35^, ^36^, ^37^ Many key oncogenes in tumour cells are driven by super‐enhancers, which play a key role in the development of tumours.^37^, ^38^ As a result, super‐enhancers have diversified regulation of target genes, such as promoting mRNA generation, miRNA transcription and maturation and lncRNA transcription, among others. Meanwhile, eRNA generated by super‐enhancers also plays a synergistic role in their activity.^39^, ^40^, ^41^, ^42^

Endothelial PAS domain‐containing protein 1 (EPAS1, also known as Hypoxia‐inducible factor 2‐alpha, HIF‐2α) is a cytokine containing the bHLH‐PAS domain cloned by Tian et al. in 1997.^43^ It is the main transcription factor for cells and tissues that deal with hypoxia and plays an important role in the changes to enzymes or factors caused by hypoxia. EPAS1 is mainly expressed in the placenta, heart, lung and endothelial cells.^44^ It induces and regulates the expression of genes necessary for tumours to adapt to hypoxia, such as genes encoding vascular endothelial growth factor (VEGF), EPO, GLUT and glycolytic enzymes, thus playing an important role in vascular growth, bone marrow haematopoiesis, energy metabolism and tumorigenesis.^43^, ^44^ EPAS1 becomes ubiquitous in the human body during hypoxia and can promote the production of angiogenesis factors such as VEGF to promote tumour neovascularization, enhance the malignant behaviour of tumour cells and resist radiotherapy.^45^ Studies have shown that the imbalance of EPAS1 expression is related to various tumours such as rectal cancer, pheochromocytoma, neuroblastoma, renal cancer and non‐small‐cell lung cancer. However, it can be expressed in various tumour tissues or cells and regulate their biological behaviours.^46^, ^47^, ^48^, ^49^, ^50^, ^51^ EPAS1 promotes ferroptosis in clear cell carcinoma by upregulating hypoxia‐induced lipid droplet‐associated (HILPDA/HIG2). As a regulator of enriched lipids, HILPDA, which contains polyunsaturated fatty acyl side chains, promotes ferroptosis.^5^, ^52^, ^53^ Therefore, as an HFRG, EPAS1 plays an important role in cervical cancer.

In this study, we used the GEO data set to screen for HFRGs through machine learning and identify hub genes. The expression and role of EPAS1, an HFRG, in cervical cancer were studied. The regulatory mechanisms of EPAS1 were studied using ceRNAs and super‐enhancers.

## 2 MATERIALS AND METHODS

### 2.1 Data collection

The cervical cancer gene expression data sets GSE7803, GSE63514 and GSE138080 were downloaded from the GEO database (https://www.ncbi.nlm.nih.gov/geo/). The GSE7803 data set, including 24 normal and 28 cancer samples, was derived from the GPL96 platform in the [HG‐U133A] Affymetrix Human Genome U133A Array. The GSE63514 data set, including 10 normal and 24 cancer samples, was derived from the GPL570 platform of the [HG‐U133_Plus_2] Affymetrix Human Genome U133 Plus 2.0 Array. The GSE138080 data set, including 10 normal samples and 10 cancer samples, was derived from the GPL4133 platform of the Agilent‐014850 Whole Human Genome Microarray 4 × 44 K G4112F. Hypoxia‐related gene sets were acquired from the Molecular Signatures Database (MSigDB; https://www.gsea‐msigdb.org/gsea/msigdb/index.jsp). Seven priority hypoxia‐related gene sets were eventually determined: hallmark hypoxia, reactome cellular response to hypoxia, Buffa hypoxia, Harris hypoxia, Mizukami hypoxia up, Mizukami hypoxia down and winter hypoxia metagenes. Ferroptosis‐related gene sets were acquired from the FerrDb V2 Database (http://www.zhounan.org/ferrdb/current/). We downloaded driver, suppressor and marker genes for ferroptosis. After removing duplicates, 493 hypoxia‐related and 464 ferroptosis‐related genes were identified for subsequent analyses.

### 2.2 Identification of differentially expressed genes, differentially expressed HFRGs and differentially expressed HRGs

Upon R (4.3.1) ‘limma’ package, differentially expressed genes (DEGs) from the merge data were with the cut‐off criteria of |logFC| ≥ 0.3, *p*.Val. <0.05. We constructed a linear model fitted using lmFit. DEGs were analysed using the eBayes function. Receiver‐operating characteristic curve (ROC) analyses were performed using the function ‘roc’ in the R package ‘pROC’. We then identified differentially expressed HFRGs by intersecting GEO data sets, Hypoxia‐ and Ferroptosis‐related genes and visualizing them using the Venn diagram with the R package ‘VennDiagram’.

### 2.3 Hub genes selection by least absolute shrinkage and selection operator (LASSO) and support vector machine‐recursive feature elimination (SVM‐RFE) algorithms

Characteristic genes were screened using two machine‐learning algorithms, LASSO and SVM‐RFE. As a regression analysis algorithm, LASSO (least absolute shrinkage and selection operator) can identify genes significantly associated with different samples. R software (4.3.1) package ‘glmnet’ was used to applies regularization for variable selection. Another widely used supervised machine‐learning protocol for classification and regression support vector machine‐recursive feature elimination (SVM‐RFE), was used to find the best variables by deleting the SVM‐generated eigenvectors. SVM‐RFE algorithm based on ‘e1071,’ ‘kernlab,’ and ‘caret’ packages.^54^ Overlapping genes between the LASSO and SVM‐RFE algorithms were used for further analyses. Statistical significance was defined as a two‐sided *p* value <0.05.

### 2.4 Functional enrichment analysis

Molecular Signatures Database (MSigDB) Hallmark Gene Sets and Kyoto Encyclopedia of Genes and Genomes (KEGG) pathways were used for pathway enrichment analysis. Gene set enrichment analysis (GSEA) was used to explore variations in pathway activities between the two groups. The significance threshold was set at an adjusted *p* < 0.05. R package ‘ClusterProfiler’ was used to visualize the results.

### 2.5 Immune infiltration

We evaluated the infiltration enrichment of immune cell types using the single‐sample GSEA (ssGSEA) method in the R package GSVA [1.44.5]. Wilcoxon rank‐sum test and Spearman's correlation were used to evaluate the association between immune cell infiltration and EPAS1 mRNA expression. The correlation between EPAS1 expression levels and immune cell infiltration was evaluated using R software (4.3.1) GSVA package (1.44.5) and visualized using ggplot2[3.3.6].

### 2.6 Clinical sample collection and ethical considerations

Cervical cancer tissues (*n* = 23) and normal cervical tissues (*n* = 20) were collected from the Second Affiliated Hospital of Zhengzhou University from March 2020 to March 2021. Every patient did not receive any pre‐operation treatment. This study was performed in accordance with the Helsinki Declaration and approved by the ethics committee of the Second Affiliated Hospital of Zhengzhou University.

### 2.7 Cell culture and transfection

The EPAS1 overexpressed lentivirus was synthesized by HanBio Technology. Human cervical cancer cell lines (HeLa and SiHa cells) and 293T cells were obtained from the National Collection of Authenticated Cell Culture (NCACC, Shanghai, People's Republic of China). Cells were cultured in DMEM (Hyclone) supplemented with 10% fetal bovine serum (FBS) (Gibco). The cells were maintained in a humidified incubator at 37°C with 5% CO_2_.

### 2.8 RNA extraction and cDNA synthesis

Total RNAs of human cells were extracted using TRIzol (Invitrogen) according to the manufacturer's instructions and treated with RQ1 DNase (Promega) to remove DNA. The quality and quantity of the purified RNA were determined by measuring the absorbance at 260 nm and 280 nm (A260 and A280) using a SmartSpec Plus Spectrophotometer (Bio‐Rad Laboratories, Inc). RNA integrity was further verified by electrophoresis using a 1.5% agarose gel. All RNA samples were stored at −80°C for future use. According to the manufacturer's instructions, reverse transcription reactions were carried out using the ReverTra Ace qPCR RT Kit (TOYOBO Life Science, Shanghai, People's Republic of China).

### 2.9 Quantitative real‐time‐polymerase chain reaction (qRT‐PCR)

Expression levels of EPAS1 mRNA were detected by qRT‐PCR. The human GAPDH gene was used as a control. Specific primers were designed based on cDNA sequences. Primer sequences were as follows: EPAS1, GCGCTAGACTCCGAGAACAT (Forward), TGGCCACTTACTACCTGACCCTT (Reverse); GAPDH ACCCACTCCTCCACCTTTGACG (Forward), TCTCTTCCTCTTGTGCTCTTG (Reverse). The qRT‐PCR was performed on a Bio‐Rad S1000 with Bestar SYBR Green RT‐PCR Master Mix (TOYOBO). PCR conditions consisted of denaturing at 95°C for 1 min and 40 cycles of denaturing at 95°C for 15 s, followed by annealing and extension at 60°C for 30 s. Relative gene expression was calculated using the Livak and Schmittgen 2^−ΔΔCt^ method. PCR amplifications were performed in triplicate for each sample.

### 2.10 Western blot (WB)

Protein samples were electrophoresed in 10% sodium dodecyl sulfate‐polyacrylamide gel electrophoresis gels, and resolved proteins were electro‐transferred onto polyvinylidene difluoride membranes (Millipore) in a transferring buffer (25 mM Tris, 0.2 M glycine and 25% methanol). After blocking with 5% skimmed milk, the membranes were incubated with anti‐EPAS1 (Abcam, Ab109616, 1:1000 dilution) and anti‐GAPDH antibodies (Abcam, Ab9485, 1:2500 dilution), followed by incubation with appropriate horseradish peroxidase (HRP)‐conjugated secondary antibodies (Abcam, Ab205718, 1:30000 dilution). An enhanced chemiluminescence ECL kit (ASPEN) was used to visualize the bands.

### 2.11 Flow cytometry assay for cell apoptosis detection

HeLa cells were grown on six‐well plates and transfected when the cells reached the log growth phase. After transfection for 48 h, cells were harvested, and the Solarbio apoptosis detection kit (Solarbio) was utilized to monitor the apoptotic cells.

### 2.12 Dual‐luciferase reporter analysis

Dual‐luciferase reporter was used to test the correlation between metastasis associated lung adenocarcinoma transcript 1(MALAT1), miR‐182‐5p and EPAS1. Using lipofectamine‐mediated gene transfer, pmirGLO and pmirGLO‐ATB or pmirGLO‐MALAT1‐mut (miR‐182‐5p) were co‐transfected with miR‐182‐5p mimic (or miR‐NC) into HeLa cells. Using lipofectamine‐mediated gene transfer, pmirGLO and pmirGLO‐EPAS1 were transfected into HeLa cells. The relative luciferase activity was normalized to Renilla luciferase activity 48 h after transfection.

The dual‐luciferase reporter gene was also used to detect the core enhancers in the super‐enhancer of EPAS1(Table S2). The pGL3‐basic, pGL3‐promoter, pGL3‐E1‐promoter, pGL3‐E2‐promoter, pGL3‐E3‐promoter, pGL3‐E4‐promoter and pGL3‐E5‐promoter were individually transfected into 293T cells by a plasmid. Exactly 48 h after transfection, the relative luciferase activity was normalized to Renilla luciferase activity.

### 2.13 CCK8 assay

The cells were digested with trypsin and inoculated as 10,000 cells per 96‐well plate. They were then cultured at 37°C in a 5% CO_2_ incubator for time intervals of 0 h, 24 h, 48 h and 72 h. A 10 μL CCK8 solution (Solarbio, People's Republic of China) was added to each hole, and a blank control was set. Incubation continued in the cell incubator for 2 h, and absorbance was measured at 450 nm with a microplate reader.

### 2.14 Transwell chamber assay

Cell invasion ability was evaluated using Transwell chambers pre‐coated with Matrigel. A total of 1 × 10^4^ cells were inoculated into the top chamber and incubated at 37°C with 5% CO_2_ for 48 h. After cells on top of the filter were removed, cells on the bottom were fixed in 4% paraformaldehyde and stained with 1% crystal violet (Beyotime). The invaded cells were counted on a microscope.

### 2.15 Wound healing assay

About 5 × 10^5^ cells were inoculated in six‐well plates, and they stuck to the wall and scribe in the hole. The cells were thrice washed with PBS, and a fresh medium was added. The cells were cultured at 37°C in a 5% CO_2_ incubator and photographed and recorded under the microscope at intervals of 0 h, 24 h and 48 h.

### 2.16 Super‐enhancer analysis

We used published H3K27ac ChIP‐SEQ data to analyse the super‐enhancer of cervical cancer HeLa cells and obtained the complete HeLa cell super‐enhancer map (Table S2). The brief analysis process was as follows: (1) The original H3K27ac chip‐SEQ files of HeLa cells were retrieved and downloaded from the GEO database, and the repeated data of two HeLa cells in the same study were selected as data sources (Table S2). (2) The downloaded data were filtered. (3) The reference genome of the HG38 version was selected to compare clean data obtained after filtering, and statistical comparison information was obtained. (4) Peak calling for BAM files using Model‐based Analysis of ChIP‐Seq software (MACS2) was done. (5) Using the ROSE algorithm, enhancers from peak data obtained by peak calling were identified, the enhancers distributed in the range of 12.5 kb were stitched, and the stitched enhancer was obtained. All suture enhancers were sorted according to the signal values, and the threshold values of normal enhancers and super‐enhancers were defined. (6) Using Homer software and based on the location information of the super‐enhancer, the gene closest to the TSS corresponding to the super‐enhancer was found and used as the associated gene of the super‐enhancer.

### 2.17 Statistical analysis

The statistical analysis was performed using R (4.3.1) and GraphPad Prism 10. The Wilcoxon signed‐rank test was used to compare the differences between the two groups. The correlations of gene expression and immune cells were determined by Spearman correlation and statistical significance. All tests were two‐sided; a p value of less than 0.05 was considered statistically significant.

## 3 RESULTS

### 3.1 Screening for HFRGs in cervical cancer

**FIGURE 1:**
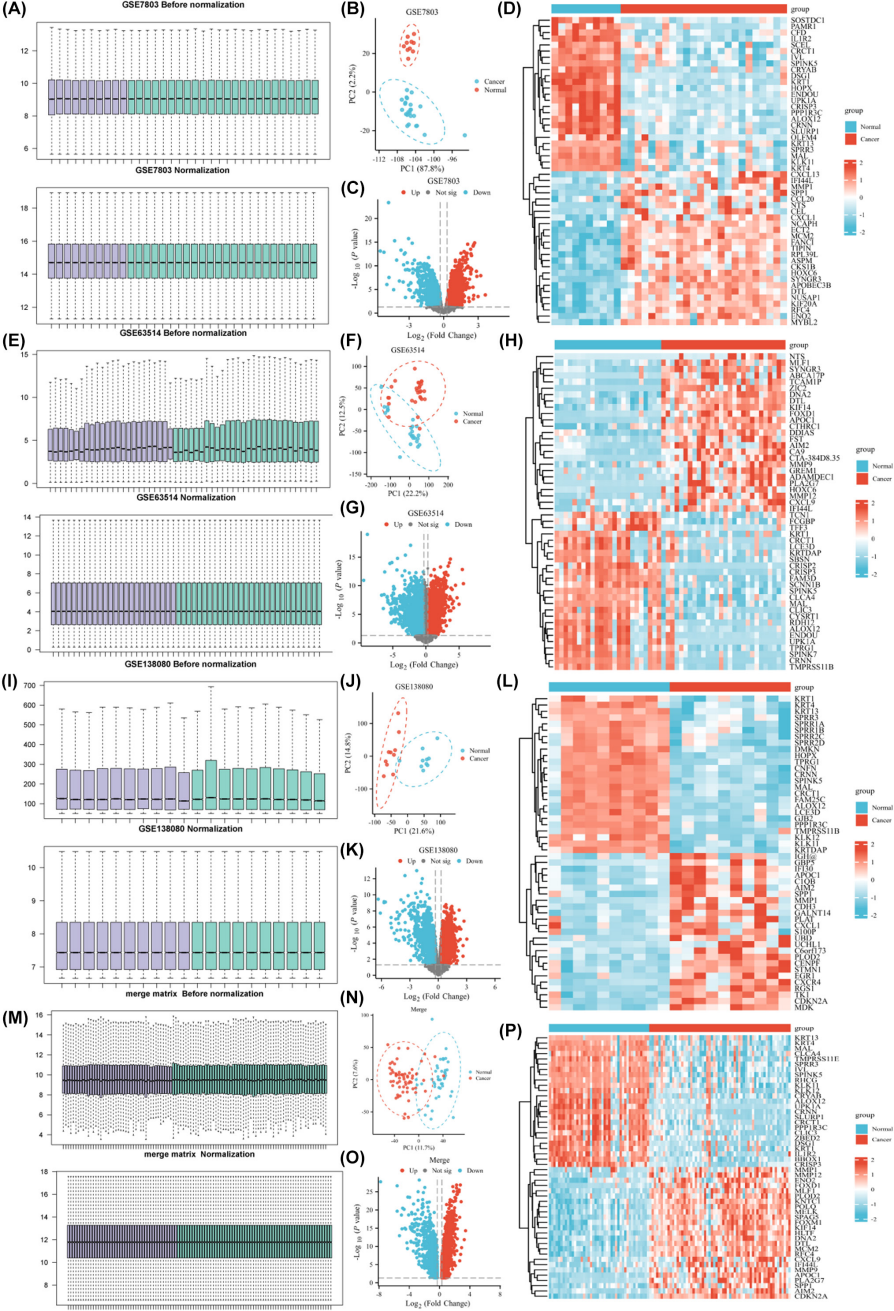
Quality control of GEO datasets. (A) The processed data boxplot before normalization and after normalization of GSE7803, (B) The PCA plot of GSE7803, (C) The volcano plot of DEGs of GSE7803, (D) The heatmap of DEGs of GSE7803, (E) The processed data boxplot before normalization and after normalization of GSE63514, (F) The PCA plot of GSE63514, (G) The volcano plot of DEGs of GSE63514, (H) The heatmap of DEGs of GSE63514, (I) The processed data boxplot before normalization and after normalization of GSE138080, (J) The PCA plot GSE138080, (K) The volcano plot of DEGs of GSE138080, (L) The heatmap of DEGs of GSE138080, (M) The processed data boxplot before normalization and after normalization of Merged dataset, (N) The PCA plot of Merged dataset, (O) The volcano plot of DEGs of Merged dataset, (P) The heatmap of DEGs of Merged dataset.

We first normalized the three data sets, GSE7803, GSE63514 and GSE138080. The processed data boxplot show that the medians of each sample were almost the same (Figure 1). The PCA plots showed good distinction between the normal and cancer samples. We set the cut‐off criteria of |logFC| ≥ 0.3 and *p* value <0.05. Subsequently, we plotted a volcano map and heatmap of the DEGs for the three data sets. Merging and normalizing the three data sets (Figure 1), and 1895 upregulated genes and 1687 downregulated genes were identified.

**FIGURE 2:**
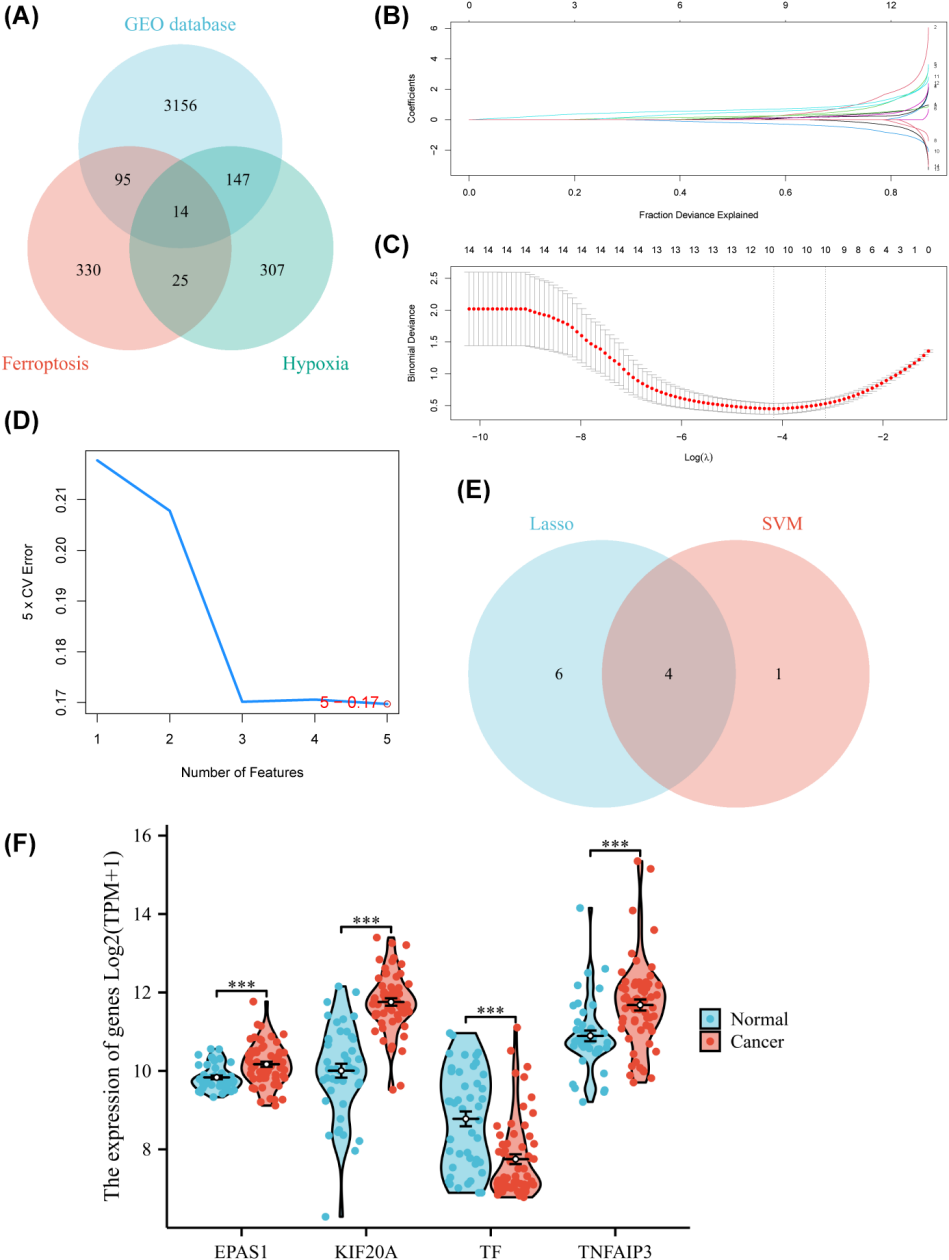
Screen the HFRGs. (A) The Venn diagram of GEO datasets, the hypoxia related‐ gene sets and the ferroptosis related‐ gene sets, (B) and (C) The results of Lasso analysis, (D) The results of SVM‐RFE analysis, (E) The Venn diagram of Lasso and SVM‐RFE, (F) The expression of four hub genes in merged GEO datasets. ***p < 0.001.

After an intersection analysis of the GEO, HFRG sets, 14 HFRGs were obtained (Figure 2A). These genes were kinesin family member 20A (KIF20A), solute carrier family 16 member 1(SLC16A1), transferrin receptor (TFRC), NEDD4‐like E3 ubiquitin protein ligase (NEDD4L), spermidine/spermine N1‐acetyltransferase 1 (SAT1), ferritin light chain (FTL), transferrin (TF), TNF‐α‐induced protein 3 (TNFAIP3), enolase 3 (ENO3), EPAS1, heme oxygenase 1(HMOX1), siah E3 ubiquitin protein ligase 2 (SIAH2), thioredoxin (TXN), ZFP36 ring finger protein (ZFP36), respectively.

Subsequently, we utilized two algorithms, LASSO and support SVM‐RFE, for machine‐learning analysis (Figure 2B–D). Intersection of the results obtained from the two machine‐learning algorithms yielded four hub genes: EPAS1, KIF20A, TF and TNFAIP3 (Figure 2E). The results showed significant differences in the expression of these four genes between the normal and tumour groups (Figure 2F).

### 3.2 EPAS1 is highly expressed in cervical cancer tissues

**FIGURE 3:**
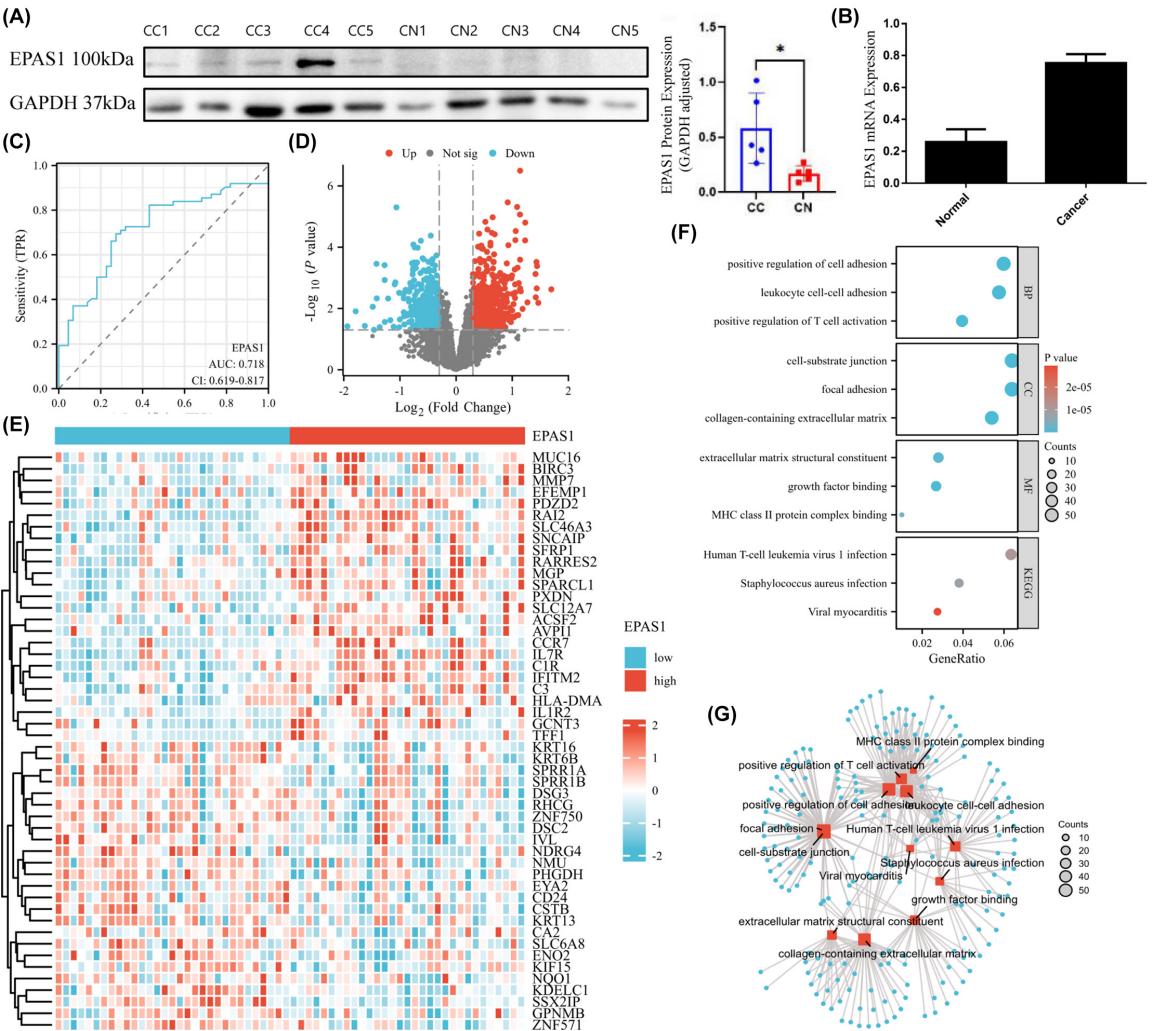
Expression of EPAS1 and GO and KEGG analysis. (A) WB showed high expression of EPAS1 in cervical cancer tissues, (B) qPCR showed that EPAS1 was significantly upregulated in cervical cancer tissues, (C) analysis of the diagnostic performance of EPAS1 by receiver‐operating characteristic (ROC) curve analysis, (D) the volcano plot of DEGs between EPAS1 high‐expression group and EPAS1 low‐expression group, (E) The heatmap of DEGs between EPAS1 high‐expression group and EPAS1 low‐expression group, (F) and (G) GO and KEGG analyses of DEGs between EPAS1 high‐expression group and EPAS1 low‐expression group *p < 0.05.

We took EPAS1 as the research target. The WB assay showed that EPAS1 was highly expressed in cervical cancer tissues (*t* = 2.816, *p* = 0.023) (Figure 3A). We detected 23 cervical cancer tissues and 20 normal cervical tissues by qPCR, and the results showed that EPAS1 mRNA was highly expressed in cervical cancer tissues (*F* = 34.910, *p* < 0.001) (Figure 3B).

ROC shows that EPAS1 has definite diagnostic values (AUC = 0.718) (Figure 3C). We divided the merged data set into high‐ and low‐expression groups based on the expression level of EPAS1. A differential expression analysis was performed. We have identified 4388 high‐expression genes and 4390 low expression genes (|logFC| ≥ 0.3, *p* < 0.05). A volcano map of the DEGs is shown in Figure 3D. The heatmap of top 25 high‐expression genes and the top 25 low‐expression genes is shown in Figure 3E.

### 3.3 GO enrichment and KEGG signalling pathway enrichment analysis

We extracted the DEGs mentioned above and performed GO and KEGG enrichment analysis. The pathways were arranged in ascending order based on the FDR values and visualized. These processes include leukocyte cell–cell adhesion, positive regulation of T cell activation, positive regulation of cell adhesion, focal adhesion, cell‐substrate junction, collagen‐containing extracellular matrix, growth factor binding, xtracellular matrix structural constituent, MHC class II protein complex binding, Staphylococcus aureus infection, human T cell leukaemia virus 1 infection, Viral myocarditis (Figure 3F,G) (Table S1).

### 3.4 Results of GSEA analysis of genes

**FIGURE 4:**
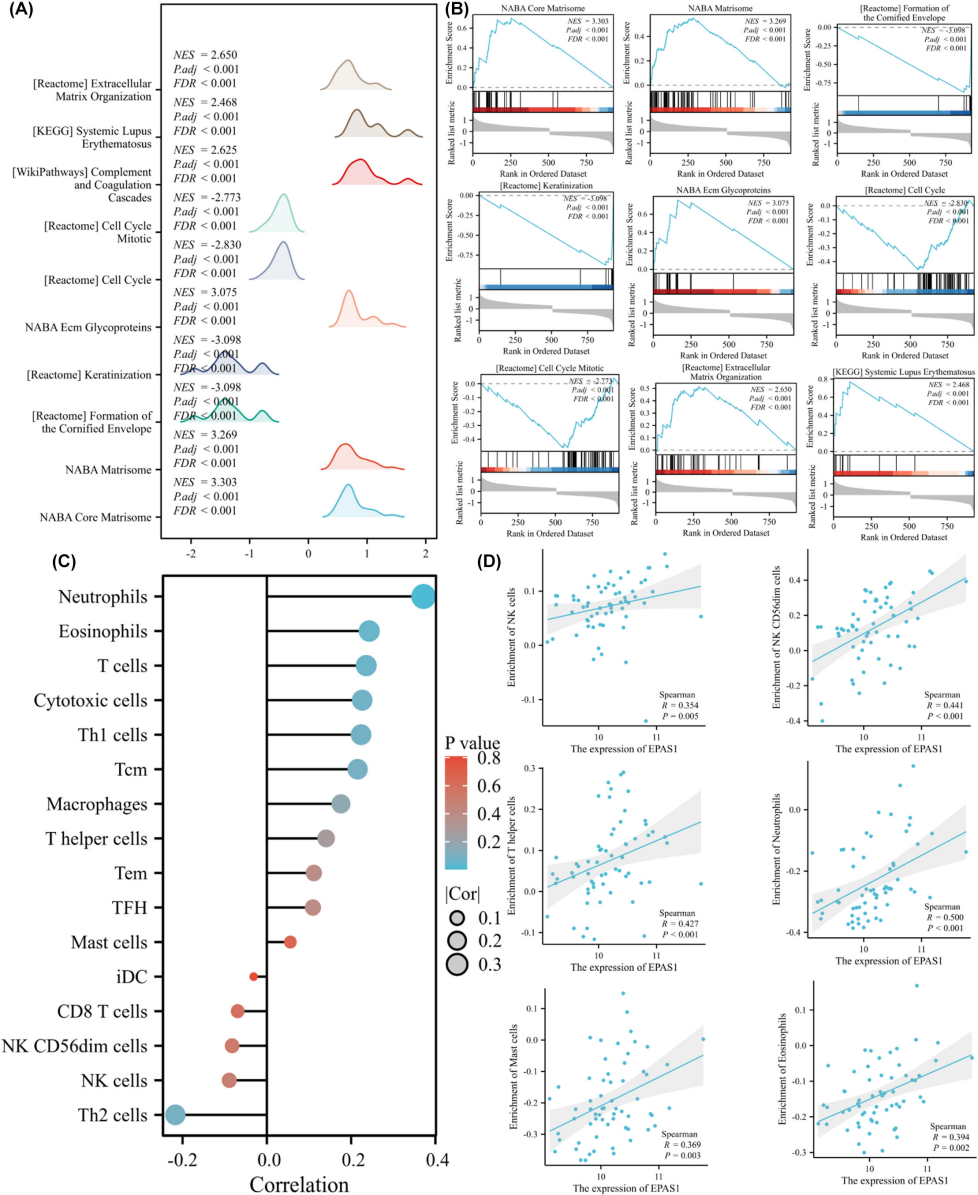
GSEA analysis and immune infiltration analysis. (A,B) The pathways enriched in EPAS1 high‐expression group and EPAS1 low‐expression group according to the GSEA, (C,D). Relationships between EPAS1 expression levels and immune cell infiltration in merged GEO data set.

To predict the function of the DEGs, we performed GSEA and found that the most 10 enriched gene sets were associated with NABA_CORE_MATRISOME, NABA_MATRISOME, REACTOME_FORMATION_OF_THE_CORNIFIED_ENVELOPE, REACTOME_KERATINIZATION, NABA_ECM_GLYCOPROTEINS, REACTOME_CELL_CYCLE, REACTOME_CELL_CYCLE_MITOTIC, WP_COMPLEMENT_AND_COAGULATION_CASCADES, KEGG_SYSTEMIC_LUPUS_ERYTHEMATOSUS and REACTOME_EXTRACELLULAR_MATRIX_ORGANIZATION (Figure 4A,B) (Table S1).

### 3.5 Correlation between EPAS1 expression and immune cell infiltration

We used ssGSEA to analyse the relationship between EPAS1 mRNA expression and immune cell infiltration. The correlation between immune cell infiltration and EPAS1 mRNA expression is shown in Figure 4C. The EPAS1 mRNA expression was found to be positively correlated with NK cells (*R* = 0.354, *p* = 0.005), CD56dim cells (*R* = 0.441, *p* < 0.001), T helper cells (*R* = 0.427, *p* < 0.001), Neutrophils (*R* = 0.500, *p* < 0.001), Mast cells (*R* = 0. 369, *p* = 0.003) and Eosinophils (*R* = 0.394, *p* = 0.002) (Figure 4D).

### 3.6 Effects of EPAS1 on proliferation, invasion, migration and apoptosis

**FIGURE 5:**
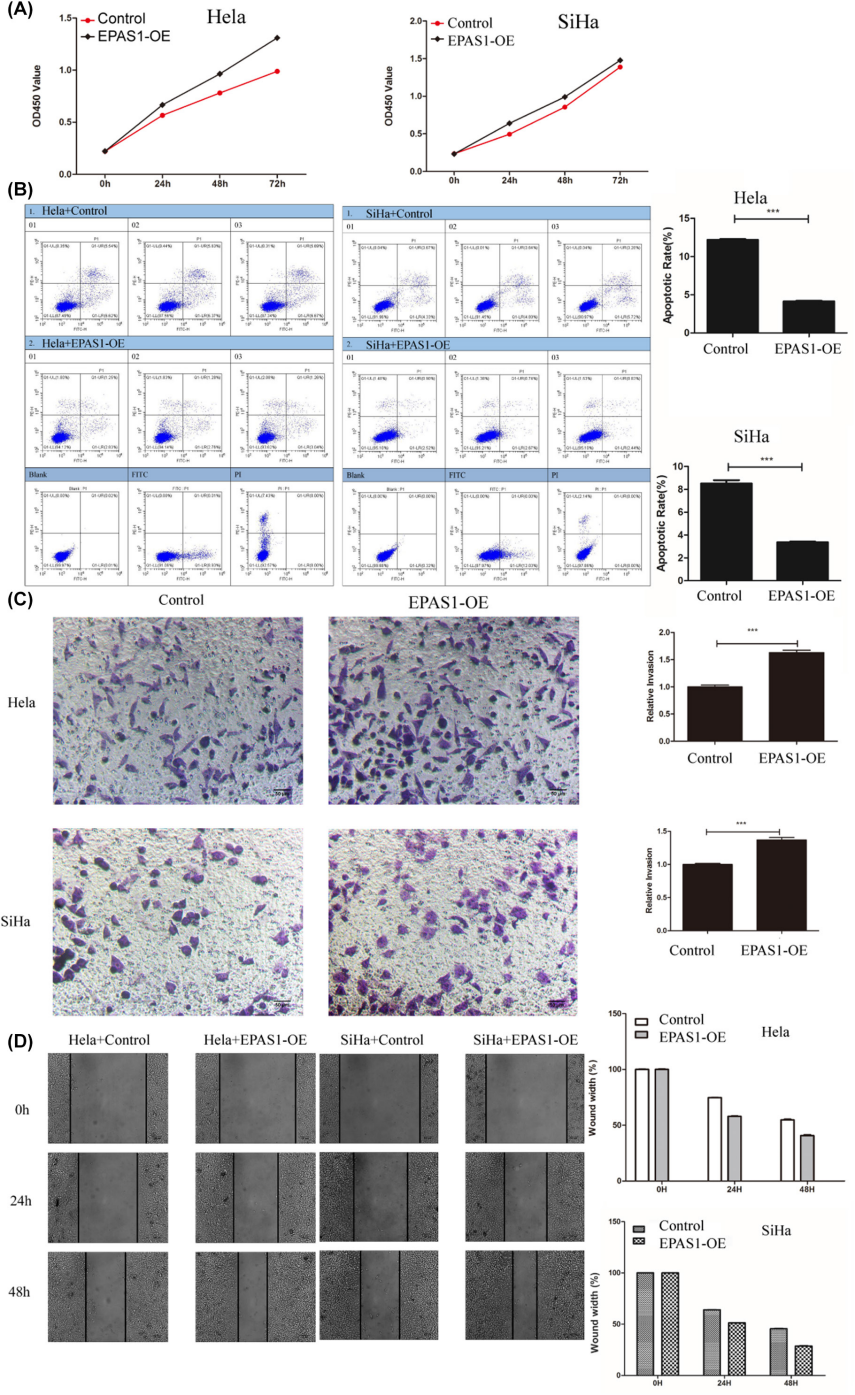
Effects of EPAS1 on proliferation, invasion, migration and apoptosis. (A) The CCK8 assay showed that cell proliferation was significantly enhanced after overexpression of EPAS1, (B) the flow cytometry assay showed that cell proliferation was reduced after overexpression of EPAS1, (C) transwell assay showed increased invasion after overexpression of EPAS1, (D) wound healing assay showed that the migration increased after overexpression of EPAS1. ***p < 0.001.

To investigate the function of EPAS1, we constructed an EPAS1 overexpressed lentivirus and transfected it into HeLa and SiHa cell lines, respectively. The CCK8 assay showed increased proliferation of HeLa cells and SiHa cells after overexpression of EPAS1 (Figure 5A). Flow cytometry showed that apoptosis of HeLa cells and SiHa cells was reduced after EPAS1 overexpression (Figure 5B). The transwell assay showed increased invasion of HeLa cells and SiHa cells after overexpression of EPAS1, and the wound healing assay showed that the migration of HeLa cells and SiHa cells increased after overexpression of EPAS1 (Figure 5C,D). EPAS1 can promote the proliferation, migration, and invasion of cervical cancer cell lines and inhibit apoptosis.

### 3.7 MALAT1‐miR‐182‐5p‐EPAS1 mRNA axis constitutes ceRNA

**FIGURE 6:**
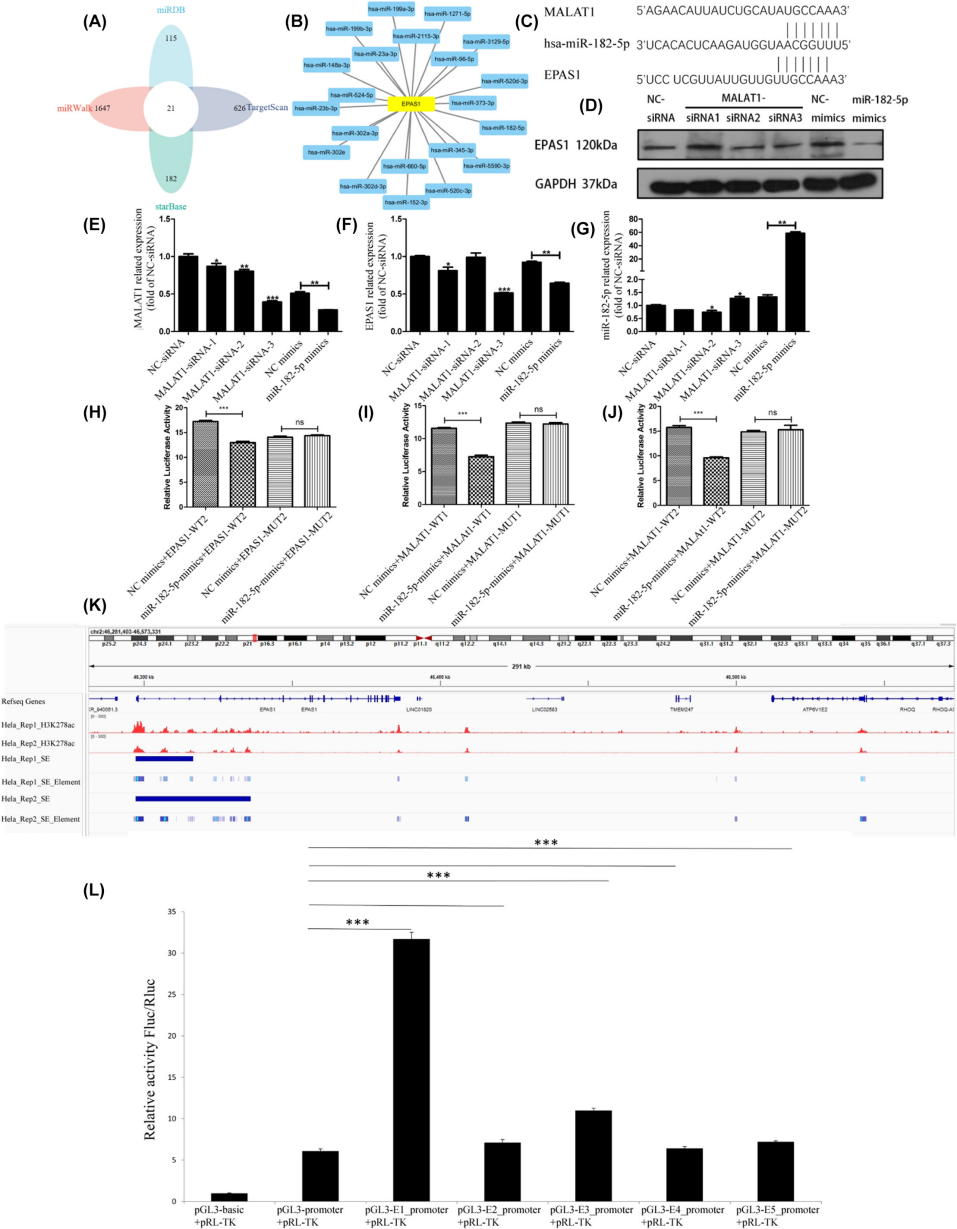
. MALAT1‐miR‐182‐5p‐EPAS1 mRNA axis and super‐enhancer analysis. (A,B) Intersection of TargetScan database, miRWalk database, starBase database and miRDB database analysis shows that EPAS1 may be the target of 21 micro‐RNAs, (C) Site of action in MALAT1‐miR‐182‐5p‐EPAS1 mRNA axis, (D) WB show that after MALAT1 knockdown, EPAS1 expression decreased. After Mir‐182‐5p overexpression, EPAS1 expression also decreased; (E–G) Synthesized three groups of MALAT1 siRNA. The results showed that MALAT1‐siRNA‐3 had an obvious effect. After overexpression of miR‐182‐5p mimics, the expression of MALAT1 decreased. After MALAT1 knockdown, EPAS1 expression decreased. After overexpression of miR‐182‐5p mimics, the expression of EPAS1 decreased. After MALAT1 knockdown, miR‐182‐5p expression increased; (H–J) Transfected with miR‐182‐5p mimics and EPAS1‐WT in Hela, the fluorescence value decreased significantly. The fluorescence value did not change significantly after EPAS1 mutation.Transfected with miR‐182‐5p mimics and MALAT1‐wt in Hela, the fluorescence value decreased significantly. The fluorescence value did not change significantly after the MALAT1 mutation; (K) Super‐enhancer of EPAS1 in HeLa cell; (L) Dual‐luciferase assay of the super‐enhancer of EPAS1. Compared with the pGL3‐basic group, the Fluc/Rluc activity of the pGL3‐promoter was significantly increased. Compared with the pGL3‐promoter group, the activity of the pGL3‐E1‐promoter and pGL3‐E3‐promoter groups was significantly increased. Compared with the pGL3‐promoter group, the pGL3‐E2‐promoter, pGL3‐E4‐promoter and pGL3‐E5‐promoter groups' activity were slightly higher. ns>0.05, *<0.05, **<0.01, ***<0.01.

Using the TargetScan database, we found that EPAS1 may be a target of 647 miRNAs. Using the miRWalk database, we found that EPAS1 may be a target of 1668 micro‐RNAs. Using the StarBase database, EPAS1 was identified as a target of 203 miRNAs. Using the miRDB database, EPAS1 was identified as the target of 136 miRNAs (Figure 6A). After analysing the intersection of the four databases, we found that EPAS1 may be a target of 21 miRNAs (Figure 6B). Using lncBase database analysis, it was found that MALAT1 may be the target of miR‐182‐5p. The action site is shown in Figure 6C.

To clarify the interaction in MALAT1‐miR‐182‐5p‐EPAS1 mRNA, we synthesized three groups of MALAT1 siRNA.

WB detection found that after MALAT1 knockdown, HIF‐2A expression decreased. After miR‐182‐5p overexpression, EPAS1 expression also decreased (Figure 6D). The results confirmed that MALAT1‐siRNA‐3 had an obvious interference effect. After MALAT1 knockdown, the expression of miR‐182‐5p increased, and the expression of EPAS1 decreased. After overexpression of miR‐182‐5p mimics, the expression of MALAT1 and EPAS1 decreased (Figure 6E–G).

We then carried out the luciferase reporter assay. When HeLa cells were transfected with miR‐182‐5p mimics and EPAS1‐WT, the fluorescence value decreased significantly. However, the fluorescence value did not change significantly after the EPAS1 mutation. When HeLa cells were transfected with miR‐182‐5p mimics and MALAT1‐wt, the fluorescence value decreased significantly. However, the fluorescence value did not change significantly after the MALAT1 mutation (Figure 6H,J). These data suggest that the MALAT1‐miR‐182‐5p‐ EPAS1 mRNA axis constitutes ceRNA.

### 3.8 Super‐enhancer of EPAS1

According to our analysis, the EPAS1 gene has a super‐enhancer in HeLa cells (Figure 6K). To study the super‐enhancer of EPAS1, we performed a dual‐luciferase assay. We constructed plasmid 1 PGL3‐promoter. The predicted super‐enhancer region was divided into four parts, and plasmids were constructed with each part's promoter region. Plasmid 2 was pGL3‐E1‐promoter (chr2: 46297348–46,299,933 + pGL3‐promoter). Plasmid 3 was pGL3‐E2‐promoter (chr2: 46305620–46,306,140 + pGL3‐promoter). Plasmid 4 was pGL3‐E3‐promoter (chr2: 46306651–46,308,013 + pGL3‐promoter). Plasmid 5 was pGL3‐E4‐promoter (chr2: 46315696–46,316,103 + pGL3‐promoter). Plasmid 6 was pGL3‐E5‐promoter (chr2: 46316165–46,316,653 + pGL3‐promoter) (Tables [Link], [Link]). Compared with the pGL3‐basic group, the Fluc/Rluc activity of the pGL3‐promoter was significantly increased, indicating that the promoter was active. Compared with the pGL3‐promoter group, the activities of the pGL3‐E1‐promoter and pGL3‐E3‐promoter groups were significantly increased, indicating that E1 and E3 elements had enhanced the promoter activities. Compared with the pGL3‐promoter group, the activities of the pGL3‐E2‐promoter, pGL3‐E4‐promoter, and pGL3‐E5‐promoter groups were slightly higher, indicating that E2, E4 and E5 elements did not significantly enhance the promoter activities. Therefore, E1 and E3 elements can significantly enhance the activity of promoters and are the core promoters of EPAS1 (Figure 6L).

## 4 DISCUSSION

One basic feature of physiological processes and pathophysiological conditions such as cancer and ischaemic diseases is hypoxia, and the main regulators of adaptive response to hypoxia are HIF‐1αand EPAS1(EPAS1). HIF‐induced genes promote characteristic tumour behaviour. The basic adaptation to continuous hypoxia is to inhibit mitochondrial respiration, induce glycolysis and affect the process of oxidative phosphorylation.^55^

EPAS1 is the main effector gene that helps cells adapt to chronic hypoxia. Under hypoxia, EPAS1 can promote the production of angiogenesis factors such as VEGF factor to promote tumour angiogenesis and enhance the malignant behaviour of tumour cells. EPAS1 overexpression can reduce cisplatin sensitivity by excessive autophagy in cervical cancer.^56^

Cancer cells accumulate high levels of iron and ROS to adapt to the high metabolic activity and rapid proliferation. Metabolic reprogramming is associated with sensitivity to acquired ferroptosis.^57^ Ferroptosis also plays an important role in the malignant biological behaviour and treatment of cervical cancer.^26^ circLMO1 suppresses cervical cancer growth and metastasis via ferroptosis.^58^ Ferroptosis, which is regulated by the Cdc25A/PKM2/ErbB2 pathway, may serve as a therapeutic target in cervical cancer.^27^ Cervical cancer (CC) progression is closely linked to iron regulation and oxidative stress.^26^

Hypoxia is also closely associated with ferroptosis. Hypoxia induces resistance to ferroptosis in cervical cancer cells.^59^ D‐mannose alleviates osteoarthritis progression via EPAS1 through ferroptosis.^60^ EPAS1 promotes ferroptosis via hypoxia‐related genes in clear cell carcinomas.^52^, ^53^ Therefore, studying HFRGs is important for clarifying the malignant biological behaviour of cervical cancer.

In this study, we identified four hypoxia‐ and ferroptosis‐related hub genes by machine‐learning algorithms. Our study showed that EPAS1 was highly expressed in cervical cancer. We found that the MALAT1‐miR‐182‐5p‐EPAS1 axis regulated EPAS1. EPAS1 could promote the proliferation, invasion, and metastasis of cervical cancer and inhibit the apoptosis of cervical cancer cells. We also analysed EPAS1 and immune cell infiltration of cervical cancer. It was found that EPAS1 was related to various immune cell infiltrations.

There are associated super‐enhancers upstream of EPAS1. Subsequently, we confirmed through experiments that the E1 and E3 elements of the super‐enhancers are the core elements that regulate EPAS1 expression. The identification of the super‐enhancers upstream of EPAS1 helps us to explore the mechanism of occurrence and development of cervical cancer, and further deepen our understanding of the role of super‐enhancers in cervical cancer. Meanwhile, this super‐enhancer may also be a potential target for the treatment of cervical cancer.

However, this study had some limitations. The mechanism by which EPAS1 combines hypoxia and ferroptosis needs to be further clarified. Further research is needed to identify other genes associated with HFRGs in cervical cancer. Future research may focus on the following two aspects: the pathways involved by EPAS1 in hypoxia and ferroptosis, and drugs targeting the super‐enhancers upstream of EPAS1.

In conclusion, we identified that EPAS1 is a hypoxia‐ and ferroptosis‐related hub gene. EPAS1 is highly expressed in cervical cancer tissues and promotes the proliferation, invasion and migration of cervical cancer cells while inhibiting apoptosis. EPAS1 expression is regulated by the MALAT1‐miR‐182‐5P‐EPAS1 mRNA axis. Its expression is also regulated by its associated super‐enhancers, of which E1 and E3 are the core elements. Therefore, EPAS1 may be a target for medical diagnosis and treatment of cervical cancer.

## AUTHOR CONTRIBUTIONS

**Xiaoqin Lu:** Conceptualization (lead); data curation (lead); investigation (lead); methodology (lead); project administration (lead); resources (lead); software (lead); supervision (lead); validation (lead); visualization (lead); writing – original draft (lead); writing – review and editing (lead). **Wenyi Zhang:** Data curation (equal); methodology (supporting); software (equal); validation (supporting); visualization (supporting); writing – review and editing (equal). **Jingyan Zhang:** Methodology (supporting); software (supporting); visualization (supporting); writing – review and editing (supporting). **Dan Ren:** Visualization (supporting); writing – review and editing (equal). **Panpan Zhao:** Visualization (supporting); writing – review and editing (supporting). **Yanqi Ying:** Methodology (supporting); writing – review and editing (supporting).

## CONFLICT OF INTEREST STATEMENT

The authors declare no conflict of interest.

## CONSENT FOR PUBLICATION

Not applicable.

## Supporting information


Table S1.

Table S2.

## Data Availability

The data sets used or analysed during the current study are available from the TCGA GDC website (https://portal.gdc.cancer.gov/), GEO database (https://www.ncbi.nlm.nih.gov/geo/) and FerrDb V2 Database (http://www.zhounan.org/ferrdb/current/).
