# Supplementary material for: EPAS1, a hypoxia‐ and ferroptosis‐related gene, promotes malignant behaviour of cervical cancer by ceRNA and super‐enhancer
Source: J Cell Mol Med. 2024 May 9;28(9):e18361. doi: 10.1111/jcmm.18361 (PMC11081013; doi:10.1111/jcmm.18361)
Supplement: Supplementary file 2 — Table S2. [file JCMM-28-e18361-s001.docx]

H3K27ac ChIP-SEQ data to analyze the super-enhancer of HeLa cells


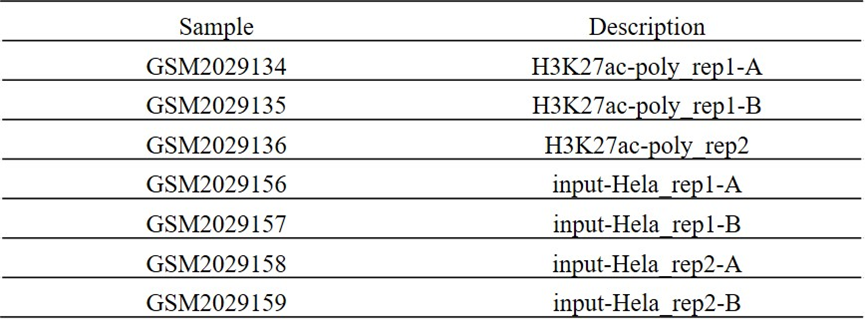


**EPAS1promoter-1 pGL3-Basic**

GGACTCAGAGGCTGTTGTGGTGAACTACTGTAGGCACCTGGAAGCCACCAAGGTGCCCTTTTCCCGCACT

CTAGTCCCAAGTGAGGGAAATAAGCCTATGGTTAAGGGAGAACTATCTACCAATTTGAACTCATTAAAAT

ACATAAATGTGTCCACTAGAAATGGCTGGCTGGAGAAGCCCAGTACCAAATCACCCCGTTCCTGAAACCC

ACCAGCCCCATGAAATAGTTCGCTAGAGAGAGATTTGCAATCTTTGAATTCGTTGACTATCAACCTGTAT

AGGTGCTCCGTGCCCCGAGATCTGAGAAAATGACAAAATACGTCTACTTCCCGGCCGGGCACAGTGACTC

ACGCCTGTAATCCCAATACTTTGAGAGGCCAAGTTGGGCAGATCACTTGAGGTCAGGAGTTTGAGACCAG

CCTGGCCAACATGGTAAAACTCCCGTCTCTACTAAAAATACAAAAATTAGCTGAGGGTGGTGGTGGGCGC

CTTTAATTCCAGTTACTCCGGAGGCTGAGCCACAAGAATCGCGGGAAGCGGAGGTTGCAGTGAGTCGAGA

TGGGGCCACAGCACTCCAGCCTGGGCGACAGAGTGGGACTCAGTCTCAAAAAAAAAAAAAAAGTATTTCC

CAATTCTTCGTCAGTTTTTGCCCTCTCCCCACTTCGCCTTCTCCAGGCTCCTGAAAAGTATCTCCCCCAA

CCCTTTCTGTGTACACGGGGAAAACACGTGTTCATTTTTATTATGGCGCTGAAACCTACCCTTCTCTTCA

AATCGCTTCTGTAGATCGTTCTAGTATAAAATGTGGCAAGAGGCTAAAGGGAGAGATATAGACCAGTTCT

TTGGCCCTCGCTTTCCAACTTCAAGTTACACCTGTGAAACTCATGGGTCCTTCCACAGCCTTCAAAAACT

AAGGGCGTCCCCTGTCCTCTCCCCAGATGTCCCTTCCCCATCGCCGGTAGCGAGTGGGAGACAGCTCAGC

GCGGGGCAGGGGAGCACTGGGCCCGGAGATGGAAGGCAGCGTCAAAAGCGCCGCTGGAAAATCCCTGAGC

GCTAACCGTTGCCTGTGTGAGCCCTTAAATCTACAAATTTCCAACACCTGTAGCCTTTGGGTTTCCCAGG

ACTTCCATCGACCCTGGCGGCAGAGAGGGCAGGCCTGAGATGCAGTGACTTGAGGGCACATGGCCAACTC

TTGTCACTCCAAGATCACACTGGGGAACCAGACTGACTTCTCCAATTCTGAACTCGCCCCGGCCTCGGGC

GGCTCAAAGGGCCTCCTCTGCCGCATCCCCGCCAAAACCAAACCGCCTGGCACAAGCCGGTAAGCAACCA

CCCTGCTGGGAGAGGGAAGGAAGAGTAGGCGCAGCCCTAGATCAATTTCCTTGCACTGCTTCTCCCAGAC

GGTCAAGTCAGCTGCGTCCCACCGAAAAGGGCGCATCGCCCACGCCCGAAACGCAGCCGCTGGGGGCCGA

GAAATTATCCCCACCTGGCCCGAGGGCCAGGGACGCAGGAGCGCAGCAGCGTGGAGGGGCTCCGCGCTGG

CCCGGCGCTGCCCGCGGTCCTGCCCTCGTTCCAAGGGCACGGCGCCGGTACGAGGACACCGACGCTGTGG

CGCAACTGCCGTCCCCCGCAGCAATCCCGGAGCCCGGCTCCCGGCCGCCCCTCGGCCCTGCGCAGGCTGC

CTCTCCCCGACGCGGAGTCCCACCCCGCTACCCGCCGCCCAGACGACCTCATAAACAAGTCCTCGAAGTG

CGGAGGCAGGAGGCGGGGCGCAGCGCGGGGGCAGGAGGCGGGCCAGGGTCAGGGCAGAGGCTGCGGCCGC

GCGTCCCCATTGGCCGGGACGCAGTGAGCCGCCCGGAGCTCGGCGCGGGCGGGGCCTGCCGGCGCGTGCC

CGCCCACACACCCGCGCCGGTGCCCGCCCCCCGCCCTCCGCGCCCGCCCCGTGCCCGCCCCAAGCCGGCC

GACGGAGTTTTTAAAGTGGGCTGCCGGCCGCGGGAGCTTT

**EPAS1promoter-2 pGL3-Basic**

TGCCCGCCCCAAGCCGGCCGACGGAGTTTTTAAAGTGGGCTGCCGGCCGCGGGAGCTTTACACTCGCGAG

CGGACCGCCACACGGGTCCGGTGCCCGCTGCGCTTCCGCCCCAGCGCTCCTGAGGCGGCCGTACAATCCT

CGGCAGTGTCCTGAGACTGTATGGTCAGCTCAGCCCGGCCTCCGACTCCTTCCGACTCCCAGCATTCGAG

CCACTTTTTTTTTTCTTTGAAAACTCAGAAAAGTGACTCCTTTTCCAGGGAAAAAGGAACTTGGGTTCCC

TTCTCTCCGTCCTCTTTTCGGGTCTGACAGCCTCCACCCACTCCTTCCCCGGACCCCGCCTCCGCGCGCA

GGTTCCTCCCAGTCACCTTTCTCCACCCCCGCCCCCGCACCTAGCCCGCCGCGCGCCACCTTCCACCTGA

CTGCGCGGGGCGCTCGGGACCTGCGCGCACCTCGGACCTTCACCACCCGCCCGGGCCGCGGGGAGCGGAC

GAGGGCCACAGCCCCCCACCCGCCAGGGAGCCCAGGTGCTCGGCGTCTGAACGTCTCAAAGGGCCACAGC

GACAATGACAGCTGACAAGGAGAAGAAAAGGTAAGCGGGCGTCCGGGCCGATCAGGGGGCCGGTCCGAGG

CCAGGGCCGGGCTGCGCGGGGCAGGCGCGACCGAGAGTGGTTGGGAGAAGAGTGCTGAGAGGTCTTCGGA

GCTCGAGGCTCGGTTTGGAGGGCTGTGAGGGGGAGAGCATGTGCCCGGTTTGGGGGCGCGGATCAGCAGC

TTTGCAGTGGAGACTTCTGCGGCTCGGAGGAGTCGGGGATTTGCGCGCACGGCGAGGCCAGGAGGCCGAG

GGAGATGGCCTGGAGGTCGGAGGTGCTTCCGCGGTGCCTTTGAAAATCTCCCAGCCGCCCCGCAGCAGAT

TACCGTGGCCACCGGCGCTTGGAAATGTTTGTGGGTGCATGTCCTCGACTTTCTCTGGTCTCTCATTTTG

GGGCAGCATCCACGTCTCTATTTTTCTCTGGATTCGCGGAGTCCTTCCAAATGCGCTCCTGTGCCCGCGC

CGCGGCCCAAGTGGGCAAAGGGGGGCGGGAGGCGGAGAGCGGCTCAGGGACACGATCCTACCGAGGAGCC

AGGACCCTTCAGAGCGCCCCGCTGCCGGGCTCCAGACCCCACCGAAGGTTGGAGAACCCACTTCCTCGTG

CCGCACCTTGACTCTGGGGAGAGTAGGTAGTGAGTGACCTGGATTGCCCTGCGGGGGCAGATGGTTCGGT

GTGTAGGAAGCGGACGGCAACACTGGATGTCCCTGGCAGGTCCGGGTGTCTGTCTCCGAAGAGGACAGAG

AAGGGCAGCCACGATCTGCCCGCCTGCCCTCGCGAGCCTCTCGGCACTGGGTGAGAGGCAACTCTGGCCA

TTTCTTGCTGCCCTCTCGCCCTCTCCCGGCCGCTCCCAGTCCAGCCGGGCCCGGCGCTACCCGAGCGAGG

GTTCGAGCCCTCTGCGCGGCCGCGCAGAAGCAGGCAGGGCCTCAACTTCTGCAAATGTGTGCGGCTCGCC

GCCTGTCCCCTTTCTCCTCCTGTCTCCACCCGTGCGGCCCCAGTGGCCTGGAGCTTCCAGCCCCGCGCTT

GGCCGCGGCTTGGCGAGGCTATGCTGCGGGAAGCTGGAATCCAACGCGCGGCCGGCTGAACCGCCTGAGC

CGCGGGAAACCAGCGGCGGAGCGGCGGTATAGAGGTGCGAGGATGAGGAGGACCGACTCGCTAAGGGAGG

GAGGTGACTGGCCGGGAGGGCTCACTGCCGAGGTTGATTTGCTCTTGTTCCAACTTCCACTACGGGACTG

GAGCCAGAAACCGTGTATCCTCCGGTCGAAAGCAGCGGTTCCCACCTCGGGGCACCGATAAGGATTTGAT

AAACGGGAGCGAATCGCGCCTGTCCTGGCTCGGCGCCCGGGCCCTTCACTGCGGGATCGCTAGGGGAGGG

GATTGGGCCCTGCTCCCCTTTCCTGGCCGTAGTCCCCCCTTTCTCGTCACCGTCGCGCCCTCAGACCCAA

GACAAGTGAGGGAGCCCTGAGACTGCAGCCGGCACATTCCCAGGGCCCTCGGAGCAGTCCTGGGGTTCCA

TGTCTTTTGAAGGCAGCAGAGCAAGGAAAAGACTCTTAAGATTCCAGTGCGTTTTGTAAAAACCAATCAT

AATGCTAATAATGACTGAACCATCGAAAAGCAAAGTTATAGACAGCGCCTGCAGACGCTTTCGGGTCCGG

AGCCGGGGAGGGAACTTGTGTATTTTACAACTGCGATGCCTATCGGGAATGTGGCAATCGCCGAGGTGGG

GCCTGACAGTAATCTTGACAAATGTGTGAAGTGTCAGCTCCTCCTGCGCTCCTTAGCGCCGGGTTTTGTG

CAGTTTTGTACCGTATATACCGAGAAACTTGGGAGCAGGGGAAAAATGATTTCGACACACCCAGCTACAG

TACCTGGTTCGTTAGCGGACTGTTGGAAAGAGAGAAGAAGCGGGAATGGGAGCGGGAAGAACGTTGCGTA

ATTAGACTCCCAATTATTGGCGAGAGCGGCCGCTTTAAGAACCACTGTGGGGACTGCCTGCGAAAC

**EPAS1promoter-3 pGL3-Basic**

TTTGCCATGTACTCATCTGACGTTTTGTGAATTCTATAAAGCCATGGAAGTGGATTGTTAGAAGTGTCTC

ATACACCCCTAGTGACTTCTTGAACTATCCCCCCATCCCCAGCCATGGAACTCTCCTATTAAAAAAACTA

AGAGATAAGTGGTAGCAATCTTAACAAATTAGTGATTTTATCTGGTTAGGCCGTGTAATCAAACACGTGG

GCAGGAAAGTGGATCCATTATGTTGTACTCATGATTGTGGTGCTGAAGGTAACCCCCATTGCCCTGTAGG

CTGCCCCCAGAAGCAGGGGGTACAGCCAATAGTATTCTTAGAAGTTAATCAATTCTGTAACGGGTTGAGT

GTTTTGGTCTCATTTTTAGTCCGAGGTGCCCTGATGCTGGCCTGTGACTAAGGGGAGTCAGTGGCTTGTT

ATGAATAAGAGGACACATGAGCTGAATCTGAGAAAGTTCAGAAGCATTTTATAAGTTGTTTAAAATACAT

CCATTAAAGTATATGATTATTTTTTGGTAAT

**EPAS1promoter-4 pGL3-Basic**

GAGTGCCTTTAAGCACACATAAAAACTCGGTTACTTGGCTTTGTTTATGAATTCCACATAGAAACAACAG

AAAATTGTGCCTTTTGAGGAACTGCTTGAGAATCCTTCCTTCTGCATTTCAAGACAAGATTTTAAAAATC

ACATATATCAAATATATGGGGAAATGAGACACAATGTTTTTAGAGAGTGTCTAGAAATAATAGGACTGTG

CAAGTTAGTACAGCAAAATTTTGGGTCAAAGCATGGGTCATTTGACAAACTCTCTTTATGAACATTACCT

CTGCAATATTTTTCTAAATGAAAACAATCACACTTAATAGAATTAATCAGGATGGGAGCTAGAAGCATAT

AATTCACTTTCCACAGATTGGAGTAAATCCTCATAATGCCCCTTTCCTGTGTATTTAGTTAAAAAAGCAC

TCCAGTGAGATTGCTTTCGCAGTAAGTTTCTGTAAACCACTGTACCCTTGTATTTCTCTCCTGTGTTTCC

AAGAGTTCTCAGATGCTATTTTCGAATGGCAGCGTTCACTCCAGAGCTGGCTGAGGTCAACGAAGCTAAT

TTTGGTGCCTTGGTGCCCAGGGTGAAGGGAGAGAGAAGTCCTCTGCAGAGCCAAAAGGACTCTCTGGGGC

CTGGGTTTGGAGCAGTGTGGTGTTGCTGGACACACCACATTTCACCTTTAGAACCTTTATTTGTGTGCTT

GCACAACAAATGTTTACCCAGATATTAAGATAAGGAAAGTATGAAGGTCACATTTCACTGGTGACAAGAG

AGCTTCTATAAATGTAACCATATCGTTGTGCAGCGGGAGTCTGTCTTTTATTCTGTACCCGGGACTTAGG

GGGAATTATTGGTGGGTGGGGCATGGGAGTTGAGGCTCTTCTTGGTTTTCTGGCTTTCTTGCTATCACTC

TGTTTGTCTTGCCTCAGCTGATGTGCGCCTCCCATTTGGTAAGGGCCTTTGTCATGGCCAAGACATCTAA

AGAAATCCTAGTCTGTGTGGCTGGGCACTGTGTGGTCCCCTGACTCTCTGGAGTCTTCCAGAGTTCCATG

GAAATTAGTGCCCCCGTGCATTTCCCTGCCGACTGACTGGAAGGCACCCTGGCTGCTGAGATCGCTTTCC

TGGTCCCCCAGAAGAGGTCCACATGGTGCTGATCCTGCAGCTGTTTTAGGCCTCCTCAGCGGGATTGTCC

TATCCTCGGAGCACAGAGAATCTTCAGAGTGGGATAGGGGTTTCTTTTGTGGATTTGGGTTTCTCTTAGG

GATCTCCAAAGATTAGCTAGACACTCAGGGCCTCAGTAAAGACATGGGTCCTTGGTCTAGTTTTCTTCCC

AAGGGCAGCCACCATGGCAGTGGGAGTTCACCT

**EPAS1promoter-5 pGL3-Basic**

CACTCACACTGCTGGACCGAGGGCAGGATGTAGAGGGAGGGCAGGGATCTGCAAACGTCACCCAGGGGTG

CTCTGGGCTCTGAGGGTGGAGGGCAAGAGGGGCAGAGCCCCCTCAAATTCTTTGGAAGATTATAGCACAA

GGGGAGTTGGGAAGGCCCTGGGACCATGTGCACACACATCTATCTCCATGAATTGTCTCTCCTGTAAAAT

TATTTTTAAAGTTGCTAAGTTGTTTTCTTCTGATTATAAAAAATATACATGTTCCCCATAAAAAGTCATA

GTAAAAGAAGGCATGGAGATAAAAATAAAAATCACCAAAATTCCATCACCCAGAGATAACCACTGTTAAC

TCTTTGGTGAACATACTTTTCTCTATATCATGAATATTGATATATAATTACAGAATAC

**EPAS1promoter-6 pGL3-Basic**

ATAAAACGAGTCACATGATTTTTTTTTGTTTCCTCGTGCATATGAAAGTTATGTTTATATTTTAGCATTA

TGTCTAAAAAACAATGTACATACCCTTTTTTTTTTTCTTTTTGAGACACAGTTTCACTCCGTTGCACAGA

CTGGAGTGCAATGGGGCAATCTTAGCTCACTGCAACTTCCGCCTCCTGGGTTCAAGCAATTCTGGTGCCT

CAGCACCCCCGAGTAGCTGGGATTTACAGACATGTGCTGCCACACCCAGCTAATTTTTGTGTTTTTAGTA

GAGATGGGGTTTTGCCATGTTGGCCAGGCTGGTCTCGAACTACTGGCCTCAAATGATCTGCCTGCCTCAG

CCTCCCAAAGTGTTGGGATTACAGGCATGAGCCACCGCACCTGGCCAACAATGTCTATACCTTAATTTAA

AAATATTTTATTGCTAAAAGAAATGCTAACAATCATCTGAGCCTTCAGTGAGTCATAATCTTCTTACTG
